# Supplementary material for: Chemical Constituents, Anticancer and Anti-Proliferative Potential of Limonium Species: A Systematic Review
Source: Pharmaceuticals (Basel). 2023 Feb 14;16(2):293. doi: 10.3390/ph16020293 (PMC9958820; doi:10.3390/ph16020293)
Supplement: Supplementary file 1 [file pharmaceuticals-16-00293-s001.zip › pharmaceuticals-2149724-supplementary.pdf]

Supplementary Materials

Board S1 PRISMA 2020 Checklist

Section and Topic	Item #	Checklist item	Location where item is reported
**TITLE**			
Title	1	Identify the report as a systematic review.	1
**ABSTRACT**			
Abstract	2	See the PRISMA 2020 for Abstracts checklist.	1
**INTRODUCTION**			
Rationale	3	Describe the rationale for the review in the context of existing knowledge.	1-2
Objectives	4	Provide an explicit statement of the objective(s) or question(s) the review addresses.	2
**METHODS**			
Eligibility criteria	5	Specify the inclusion and exclusion criteria for the review and how studies were grouped for the syntheses.	22-23
Information sources	6	Specify all databases, registers, websites, organisations, reference lists and other sources searched or consulted to identify studies. Specify the date when each source was last searched or consulted.	22-23
Search strategy	7	Present the full search strategies for all databases, registers and websites, including any filters and limits used.	22-23; Appendix SB
Selection process	8	Specify the methods used to decide whether a study met the inclusion criteria of the review, including how many reviewers screened each record and each report retrieved, whether they worked independently, and if applicable, details of automation tools used in the process.	22-23
Data collection process	9	Specify the methods used to collect data from reports, including how many reviewers collected data from each report, whether they worked independently, any processes for obtaining or confirming data from study investigators, and if applicable, details of automation tools used in the process.	22-23
Data items	10a	List and define all outcomes for which data were sought. Specify whether all results that were compatible with each outcome domain in each study were sought (e.g. for all measures, time points, analyses), and if not, the methods used to decide which results to collect.	22-23
	10b	List and define all other variables for which data were sought (e.g. participant and intervention characteristics, funding sources). Describe any	22-23

Section and Topic	Item #	Checklist item	Location where item is reported
		assumptions made about any missing or unclear information.	
Study risk of bias assessment	11	Specify the methods used to assess risk of bias in the included studies, including details of the tool(s) used, how many reviewers assessed each study and whether they worked independently, and if applicable, details of automation tools used in the process.	22-23; Appendix SC and SD
Effect measures	12	Specify for each outcome the effect measure(s) (e.g. risk ratio, mean difference) used in the synthesis or presentation of results.	-
Synthesis methods	13a	Describe the processes used to decide which studies were eligible for each synthesis (e.g. tabulating the study intervention characteristics and comparing against the planned groups for each synthesis (item #5)).	22-23
	13b	Describe any methods required to prepare the data for presentation or synthesis, such as handling of missing summary statistics, or data conversions.	22-23
	13c	Describe any methods used to tabulate or visually display results of individual studies and syntheses.	22-23
	13d	Describe any methods used to synthesize results and provide a rationale for the choice(s). If meta-analysis was performed, describe the model(s), method(s) to identify the presence and extent of statistical heterogeneity, and software package(s) used.	-
	13e	Describe any methods used to explore possible causes of heterogeneity among study results (e.g. subgroup analysis, meta-regression).	-
	13f	Describe any sensitivity analyses conducted to assess robustness of the synthesized results.	-
Reporting bias assessment	14	Describe any methods used to assess risk of bias due to missing results in a synthesis (arising from reporting biases).	-
Certainty assessment	15	Describe any methods used to assess certainty (or confidence) in the body of evidence for an outcome.	-
**RESULTS**			
Study selection	16a	Describe the results of the search and selection process, from the number of records identified in the search to the number of studies included in the review, ideally using a flow diagram.	2; Figure 1
	16b	Cite studies that might appear to meet the inclusion criteria, but which were excluded, and explain why they were excluded.	2; Appendix SE
Study characteristics	17	Cite each included study and present its characteristics.	2-19
Risk of bias in	18	Present assessments of risk of bias for each included study.	19,

Section and Topic	Item #	Checklist item	Location where item is reported
studies			Appendix SC and SD
Results of individual studies	19	For all outcomes, present, for each study: (a) summary statistics for each group (where appropriate) and (b) an effect estimate and its precision (e.g. confidence/credible interval), ideally using structured tables or plots.	Table 1 (pp.4-7), Table 2 (pp.9-12), Table 3 (pp.14-15), Figure 2 (p.16) and Figure 3 (p.21)
Results of syntheses	20a	For each synthesis, briefly summarise the characteristics and risk of bias among contributing studies.	-
	20b	Present results of all statistical syntheses conducted. If meta-analysis was done, present for each the summary estimate and its precision (e.g. confidence/credible interval) and measures of statistical heterogeneity. If comparing groups, describe the direction of the effect.	-
	20c	Present results of all investigations of possible causes of heterogeneity among study results.	-
	20d	Present results of all sensitivity analyses conducted to assess the robustness of the synthesized results.	-
Reporting biases	21	Present assessments of risk of bias due to missing results (arising from reporting biases) for each synthesis assessed.	-
Certainty of evidence	22	Present assessments of certainty (or confidence) in the body of evidence for each outcome assessed.	-
**DISCUSSION**			
Discussion	23a	Provide a general interpretation of the results in the context of other evidence.	19-22
	23b	Discuss any limitations of the evidence included in the review.	22
	23c	Discuss any limitations of the review processes used.	22
	23d	Discuss implications of the results for practice, policy, and future research.	19-22
**OTHER INFORMATION**			

Section and Topic	Item #	Checklist item	Location where item is reported
Registration and protocol	24a	Provide registration information for the review, including register name and registration number, or state that the review was not registered.	23
	24b	Indicate where the review protocol can be accessed, or state that a protocol was not prepared.	23
	24c	Describe and explain any amendments to information provided at registration or in the protocol.	-
Support	25	Describe sources of financial or non-financial support for the review, and the role of the funders or sponsors in the review.	24
Competing interests	26	Declare any competing interests of review authors.	24
Availability of data, code and other materials	27	Report which of the following are publicly available and where they can be found: template data collection forms; data extracted from included studies; data used for all analyses; analytic code; any other materials used in the review.	-

From: Page MJ, McKenzie JE, Bossuyt PM, Boutron I, Hoffmann TC, Mulrow CD, et al. The PRISMA 2020 statement: an updated guideline for reporting systematic reviews. BMJ 2021; 372:n71. doi: 10.1136/bmj.n71

For more information, visit: http://www.prisma-statement.org/

Board S2 Search Strategies

The following search strategy was used for each electronic database (PubMed, Scopus, and Scielo). The search term used was "*Limonium*".

**PubMed**	Limonium[TIAB]
**Scopus**	TITLE-ABS-KEY(Limonium)
**Scielo**	Limonium

Note: [TIAB]: title/abstract

Table S1 Evaluation of the risk of bias by the adapted SYRCLE's tool for *in vitro* studies

Authors, year	Was the allocation sequence adequately and applied?	Were the groups similar at baseline or were they adjusted for confounders in the analysis?	Was the allocation to the different groups adequately concealed during?	Were the cell culture housed during the experiment?	Were the caregivers and/or investigators blinded from knowledge which intervention each cell culture received during the experiment?	Were culture cell selected for outcome assessment?	Was the outcome assessor blinded?	Were incomplete outcome data adequately addressed?	Are reports of the study free of selective outcome reporting?	Was the study apparently free of other problems that could result in high risk of bias?
Tang et al. 2012	Yes	Unclear	Unclear	Yes	No	Yes	No	Unclear	Unclear	Unclear
Kong et al. 2014	Yes	Unclear	Unclear	No	No	Yes	No	Unclear	Unclear	Unclear
Tang et al. 2014	Yes	Unclear	Unclear	Yes	No	Yes	No	Unclear	Unclear	Unclear
Medini et al. 2015	Yes	Unclear	Unclear	Yes	No	Yes	No	Unclear	Unclear	Unclear
Bae et al. 2016	Yes	Unclear	Unclear	Yes	No	Yes	No	Unclear	Unclear	Yes
Rodrigues et al. 2016	Yes	Unclear	Unclear	Yes	No	Yes	No	Unclear	Unclear	Unclear
Bae et al. 2017	Yes	Unclear	Unclear	Yes	No	Yes	No	Unclear	Unclear	Yes
Chen et al. 2017	Yes	Unclear	Unclear	No	No	Yes	No	Unclear	Unclear	Yes
Cordeiro 2017	Yes	Unclear	Unclear	Yes	No	Yes	No	Unclear	Unclear	Unclear
Lee et al. 2017	Yes	Unclear	Unclear	Yes	No	Yes	No	Unclear	Unclear	Yes
Sahli et al. 2017	Yes	Unclear	Unclear	Yes	No	Yes	No	Unclear	Unclear	Unclear

Al-Madhagi et al. 2019	Yes	Unclear	Unclear	No	No	Yes	No	Unclear	Unclear	Unclear
Amrani et al. 2019	Yes	Unclear	Unclear	Yes	No	Yes	No	Unclear	Unclear	Unclear
Hamadou et al. 2019	Yes	Unclear	Unclear	Yes	No	Yes	No	Unclear	Unclear	Unclear
Rodrigues et al. 2020	Yes	Unclear	Unclear	Yes	No	Yes	No	Unclear	Unclear	Yes
Tuohongerbieke et al. 2021	Yes	Unclear	Unclear	Yes	No	Yes	No	Unclear	Unclear	Yes

Evaluation of the risk of bias by the SYstematic Review Centre for Laboratory animal Experimentation (SYRCLE's) tool adapted. The evaluation is done for each study and estimates the possibility of the existence of low or high risk of bias in their results. **Note:** Adapted from Hooijmans et al. (2014); Chierrito et al. (2019).

Table S2 Evaluation of the risk of bias by SYRCLE's tool for *in vivo* studies

**Authors, year**	**Was the allocation sequence adequately and applied?**	**Were the groups similar at baseline or were they adjusted for confounders in the analysis?**	**Was the allocation to the different groups adequately concealed during?**	**Were the animals randomly housed during the experiment?**	**Were the caregivers and/or investigators blinded from knowledge which intervention each animal received during the experiment?**	**Were animals selected at random for outcome assessment?**	**Was the outcome assessor blinded?**	**Were incomplete outcome data adequately addressed?**	**Are reports of the study free of selective outcome reporting?**	**Was the study apparently free of other problems that could result in high risk of bias?**
Lellau and Liebezeit 2003	Yes	Unclear	Unclear	Yes	No	Yes	No	Unclear	Unclear	Unclear
Tang et al. 2012	No	Unclear	Unclear	Unclear	No	Yes	No	Unclear	Unclear	Unclear
Rodrigues et al. 2016	Yes	Unclear	Unclear	Yes	No	Yes	No	Unclear	Unclear	Unclear

Evaluation of the risk of bias by the SYstematic Review Centre for Laboratory animal Experimentation (SYRCLE's) tool. The evaluation is done for each study and estimates the possibility of the existence of low or high risk of bias in their results. **Note:** Adapted from Hooijmans et al. (2014).

Board S3 Studies excluded after full reading

Study (Authors, year)	Title	Reason for exclusion
Kawazoe et al. 2005	A novel drimane-type sesquiterpene from *Limonium wrightii*	Outcomes (not evaluate anticancer biological activity)
Awadh Ali et al. 2007	Screening of traditionally used endemic soqotraen plants for cytotoxic activity	Outcomes (not evaluate anticancer biological activity)
Kolumbaeva et al. 2007	Mutagenic effect of the rocket fuel component asymmetric dimethylhydrazine on rats of various ages	Outcomes (not evaluate anticancer biological activity
Daraban et al. 2013	Assessment on bioeconomical potential for medicinal plants in salty meadows from the aradului plain (W. Romania)	Outcomes (not evaluate anticancer biological activity)
Erena et al. 2014	Determination of mutagenic and cytotoxic effects of *Limonium globuliferum* aqueous extracts by Allium, Ames, and MTT tests	Outcomes (not evaluate anticancer biological activity)
Eren et al. 2015	A mutagenicity and cytotoxicity study of *Limonium effusum* aqueous extracts by Allium, Ames and MTT tests	Outcomes (not evaluate anticancer biological activity)
Eren et al. 2016	Mutagenic and cytotoxic activities of *Limonium globuliferum* methanol extracts	Outcomes (not evaluate anticancer biological activity)
Lee et al. 2017	Identification of hepatoprotective constituents in *Limonium tetragonum* and development of simultaneous analysis method using high-performance liquid chromatography	Outcomes (not evaluate anticancer biological activity)
Lovinskaya et al. 2017	Antigenotoxic activity of biologically active substances from *Inula britannica* and *Limonium gmelini*	Outcomes (not evaluate anticancer biological activity)
Eren et al. 2019	Effects of *Limonium effusum* ethanol extracts on cell proliferation and mutagenicity	Outcomes (not evaluate anticancer biological activity)
Mandrone et al. 2019	Sardinian plants with antimicrobial potential. Biological screening with multivariate data treatment of thirty-six extracts	Outcomes (not evaluate anticancer biological activity)
Hamadou et al. 2021	*Limonium duriusculum* (de Girard) Kuntze Exhibits Anti-inflammatory Effect Via NF-κB Pathway Modulation	Outcomes (not evaluate anticancer biological activity)
Ahmed et al. 1999	An anticancer tannin and other phenolics from *Limonium axaillare* (Fam. Plumbaginaceae)	Study design

		(notes)
Aniya et al. 2018	Development of bioresources in Okinawa: Understanding the multiple targeted actions of antioxidant phytochemicals	Study design<br><br>(review)
Kandil et al. 2000	A new flavonoid from *Limonium axillare*	Study design<br><br>(notes)
Masuda et al. 2002	Flow cytometric estimation on cytotoxic activity of leaf extracts from seashore plants in subtropical Japan: Isolation, quantification and cytotoxic action of (-)-	Intervention<br><br>(not evaluate crude extract, fractions, subfractions or isolated substances of *Limonium* species)
Zhang et al. 2014	Isolation and structural analysis of the polysaccharides of *Limonium bicolor* and the inhibition to Hela cell	Idiom<br><br>(Non-Roman characters)

Figure S1 Chemical structures of isolated compounds of *Limonium* species drawn by ChemDraw version 14.0.0.118

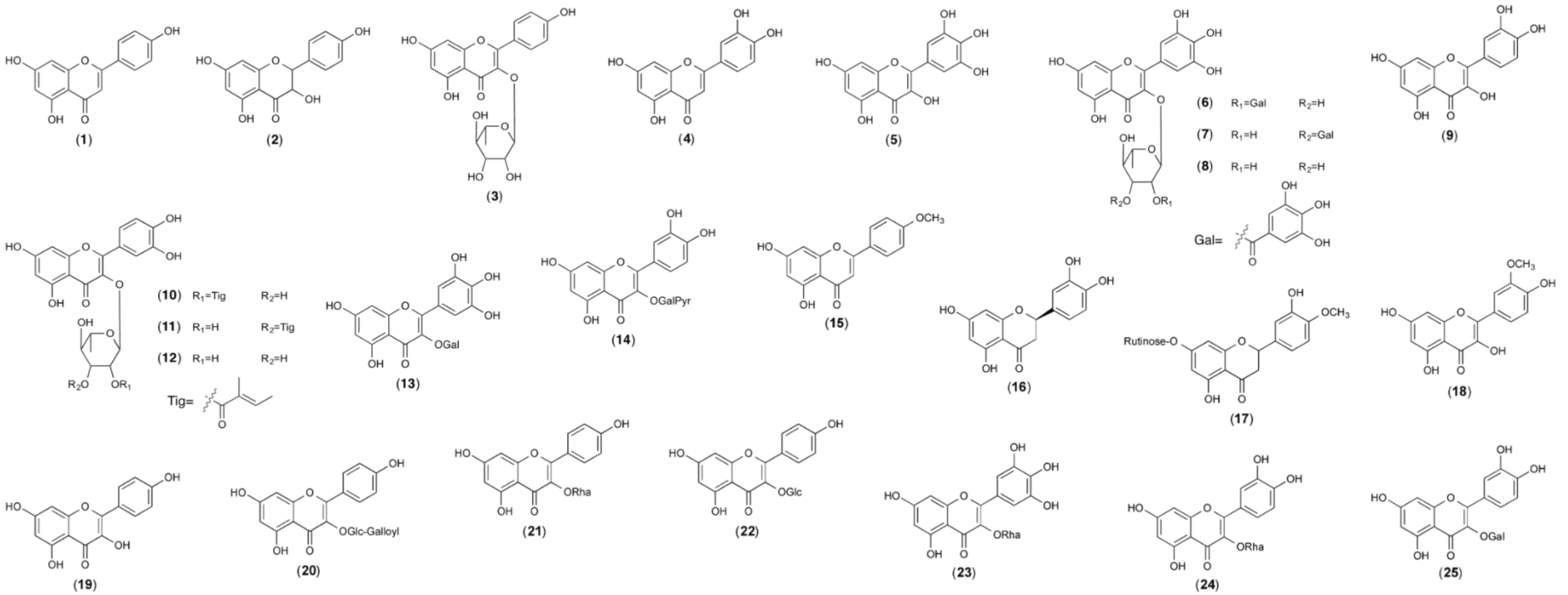

Figure S1 Continued

Figure S1 Continued

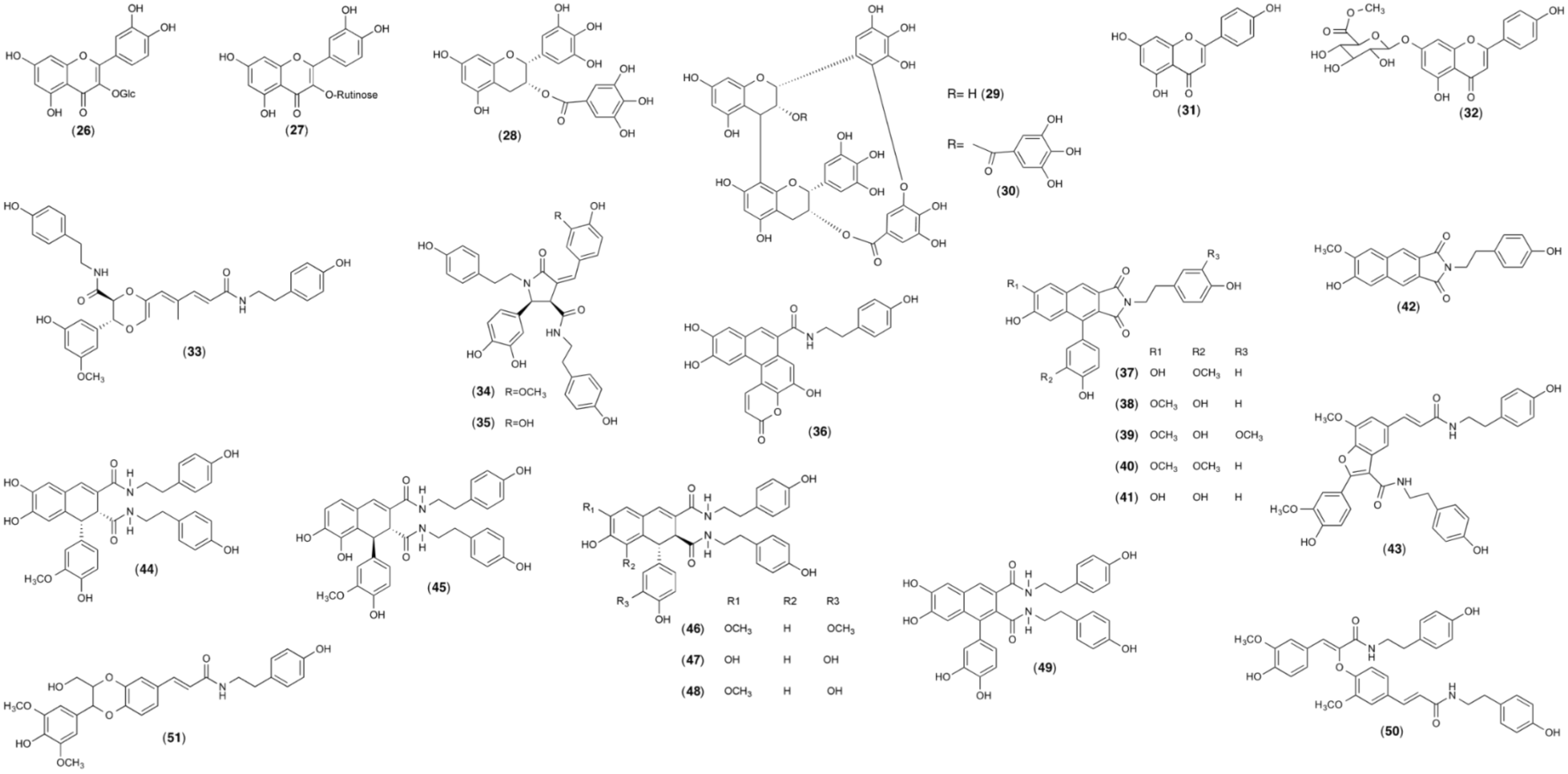

Figure S1 Chemical structures of isolated compounds 1-51 of *Limonium* species investigated for their cytotoxicities.

Table S3 Checklist for reporting data on plant material for pharmacognostic studies.

Checklist Item	Reported on page nº
**Plant Material**	
Date of plant collection (day, month, year)	
Place of plant collection (City, State, Country)	
Coordinates of plant collection (Latitude and Longitude)	
Voucher specimen number	
Name of the Herbarium code (Index Herbarium)	
Name of the person responsible for the collection and identification of the species	
The complete, correct, and accepted scientific name of the specimen *Suggestion: use of International Plant Names Index (IPNI) site (https://www.ipni.org/); *Flora do Brasil* (http://floradobrasil.jbrj.gov.br/); Royal Botanic Gardens, Kew (https://powo.science.kew.org)	
**Crude Extract Production**	
Part of the plant used	
Solvent and volume used	
Plant and solvent proportion (w/v)	
Method of extraction	
Time of extraction	
Temperature of extraction	
Yield of crude extract	
**Fraction Production**	
Solvent and volume used	
Method of extraction	
Time of extraction	
Temperature of extraction	
Yield of fraction	
